# Traditional Chinese Medicine Regulates Th17/Treg Balance in Treating Inflammatory Bowel Disease

**DOI:** 10.1155/2022/6275136

**Published:** 2022-09-15

**Authors:** Fengjiao Xie, Qin Xiong, Yilin Li, Chengjiao Yao, Ruike Wu, Qiuxiang Wang, Lihong Luo, Hongling Liu, Peimin Feng

**Affiliations:** ^1^Chengdu University of Traditional Chinese Medicine, Chengdu, Sichuan, China; ^2^Hospital of Chengdu University of Traditional Chinese Medicine, Chengdu, Sichuan, China; ^3^Department of Geriatrics of the Affiliated Hospital, North Sichuan Medical College, Nanchong, Sichuan, China

## Abstract

Inflammatory bowel disease (IBD), also known as chronic nonspecific inflammatory disease of the colon and rectum, is primarily characterized by mucopurulent bloody stools, diarrhea, abdominal pain, and tenesmus. Its cause is uncertain. IBD patients frequently experience a high rate of recurrence, a protracted treatment course, and a high risk of carcinogenesis. Additionally, the difficulty of treatment is significantly increased by these illness characteristics. Currently, the normal treatment for this illness can lessen symptoms to some amount and even meet clinical treatment requirements, but due to serious side effects, unfavorable reactions, and high costs, we need to develop better complementary and alternative medicines. A number of studies have found that the imbalance of T helper cell 17 (Th17)/regulatory T cells (Treg) contributes significantly to the occurrence and progression of IBD and that Th17/Treg balance restoration is frequently useful in the management of IBD. As a result, regulating the Th17/Treg balance has also emerged as a novel approach to treating IBD. Traditional Chinese medicine (TCM) has gained popularity in recent years due to its advantages of low side effects, a variety of targets, and multiple regulatory mechanisms. A number of studies have shown that TCM can successfully intervene in the Th17/Treg imbalance and restore it, and research on the prevention and treatment of IBD by TCM by restoring Th17/Treg has also shown promising results. The characteristics of the Th17/Treg balance and its role in the pathogenesis of IBD, as well as the role of TCM in regulating the Th17/Treg imbalance, are analyzed. The research results are expected to provide a theoretical basis for the clinical treatment and pathology mechanism research of IBD.

## 1. Introduction

Crohn's disease (CD) and ulcerative colitis (UC) are both chronic nonspecific inflammatory diseases that affect the intestines. Diarrhea, abdominal pain, bloody stools, and weight loss are the most typical clinical symptoms [1, 2]. The disease also has a number of extraintestinal symptoms, and the lesions primarily affect the joints, eyes, and skin [3]. Although the pathophysiology of Inflammatory bowel disease (IBD) is not fully understood, it is believed to be a result of complex interactions involving environmental changes, immunological dysregulation, intestinal dysbiosis, and other factors [1]. IBD is becoming more common and prevalent everywhere. It can happen at any age, and IBD patients are severely affected by the financial load [4]. According to a study in the United States, IBD patients accumulate up to $541 million in additional self-pay in treatment each year. Similarly, in a survey of the economic burden of gastrointestinal disease in the United States, IBD was ranked as the fifth most costly gastrointestinal disease in terms of annual healthcare expenditures. The financial and psychological stress on IBD patients' families is significantly increased by these high medical costs. In conclusion, the incidence of IBD is random, relapsed, and refractory and has a high incidence, high cost of treatment, and many complications making it a global public health problem [2]. IBD is a worldwide illness that has an impact on the health of millions of people. There has been a vigorous hunt for remedies for this illness in recent decades. 5-Aminosalicylates, steroids, immunosuppressive medications, and biological agents make up the majority of the pharmaceuticals used in modern medicine to treat IBD. Surgical treatment is required for some refractory cases or cases with carcinogenesis [5]. The side effects, bad responses, and high expense of various treatment methods all push us to look for further complementary and alternative therapies, even though they can somewhat ameliorate the disease and even meet the standard of clinical treatment. Numerous experimental findings indicate that the imbalance in the T helper cell 17 (Th17)/regulatory T cells (Treg) ratio in IBD patients is a significant contributor to the etiology and also has an impact on the degree of disease activity and inflammation [6]. As a result, restoring Th17/Treg equilibrium by regulation has become a cutting-edge method of treating IBD. Currently, numerous researches have established that TCM regulates the Th17/Treg balance. The effects have also been utilized for IBD, and a number of studies have been conducted with positive outcomes. TCM can be utilized as an alternative and additional treatment option for IBD treatment since it has fewer side effects, multiple targets, multiple techniques, and inexpensive rates. This paper reviews the mechanisms of recent TCM studies on IBD Th17/Treg balance, which helps to support TCM as an alternative and complementary treatment for IBD and also provides more new ideas and references for the treatment of IBD.

## 2. Role of Th17/Treg cells 

Treg and Th17 cells are both descended from CD4+ T cells. When naïve CD4+ T cells are stimulated, they can multiply, develop into other cell types, and secrete various cytokines to carry out their unique roles. In terms of action effects and developmental pathways, there is a strong link between the two, as well as significant plasticity [7, 8]. Treg cells suppress too aggressive immune responses, although Th17 cells among them can trigger inflammation and modulate immunological responses [9]. The function of Th17/Treg balance in a number of autoimmune illnesses has drawn considerable attention since studies have shown that the equilibrium created between these two is necessary for maintaining immunological homeostasis. Studies have confirmed that the balance established between these two is essential for maintaining immune homeostasis, so the role of Th17/Treg balance has received great attention in a variety of autoimmune diseases [10].

### 2.1. Source and Mechanism of Th17 Cells

Th17 cells, one of the CD4+ T cells, are crucial in autoimmune disorders [11]. Their differentiation essentially consists of three stages: Th17 cells differentiation, Th17 cells differentiation state expansion, and stable Th17 cells maturation [6]. When T cell antigen receptor/costimulatory signals, interleukin- (IL-) 6, and transforming growth factor-*beta* (TGF-*β*) stimulation are present, signaling and activator of transcription protein 3 (STAT3) is activated. Following activation, STAT3 further induces the expression of the transcription factor RAR-associated retinoic acid-related orphan receptor gamma t (ROR*γ*t), which in turn activates the transcription of IL-17 and IL-22. Second, through STAT3, IL-6 stimulates the production of IL-21, which has a favorable autocrine effect and accelerates the development of Th17 cells. Finally, IL-23R is expressed upon induction of IL-21 and renders IL-23 responsive, a cytokine that maintains the homeostasis of Th17 cell differentiation and is responsible for the stabilization of Th17 cells at later stages [12]. Th17 cells can secrete many key effector cytokines. Among them, IL-17 is one of the most important cytokines secreted by Th17 cells, which can induce the occurrence of inflammation and aggravate the damage of tissues [13]. There are six different isoforms of IL-17, but IL-17A plays a particularly important role since it can be employed by a variety of cells to stimulate the release of chemokines and cytokines [14]. Additionally, during the development of Th17 cells, the promoter region of IL-17A coordinates transcription by binding to ROR*γ*t [13]. Moreover, Th17 cells release cytokines, including IL-21 and IL-22, promote the release of proinflammatory molecules, and take involvement in the pathophysiology of autoimmune and metabolic illnesses in addition to enhancing protection against bacterial and fungal infections [10].

### 2.2. Treg Cells Source and Mechanism

Treg cells are one of the negative immunoregulatory effects of CD4+ T cells and are essential for the maintenance of immune tolerance and balance [6]. Numerous factors also affect their differentiation, and in the absence of proinflammatory cytokines, TGF-*β* induces CD4+ T cells to differentiate into Treg cells. Through the self-antigen-major histocompatibility complex on thymic antigen-presenting cells, tTreg cells are produced by receiving rather intense T cell antigen receptor stimulation. By undergoing antigenic stimulation under the effect of TGF-*β* and IL-2 in the periphery, naïve CD4+ T cells are transformed into pTreg cells. Forkhead-like protein 3 (Foxp3), CD4, CD25, and Foxp3 are all markers for T cells, whereas Foxp3 keeps its unique characteristics and functional roles [9]. To decrease naïve T cell activation and avoid the overactivity of effector T cells, Treg cells can produce the cytokines IL-10 and TGF-*β*, which inhibit the activity of a range of immune cells and, ultimately, the immune response [10]. Additionally, by directly contacting the surface of Th cells and secreting the cytokines IL-10 and TGF-*β*, Treg cells can suppress other Th isoforms such as Th1, Th2, and Th17 [15]. As a regulator of the immune system, Treg cells can maintain homeostasis and prevent the development of autoimmune diseases.

## 3. Mechanism of Th17/Treg Cells Balance in IBD

The pathogenicity of the Th17/Treg imbalance has also attracted attention and has been supported by several studies in recent years, as more studies have established that abnormalities of the intestinal mucosal immune system are a crucial element in the incidence and development of IBD [6, 14]. Th17 cells have a different distribution in the intestinal mucosa of UC and CD patients, mainly in the lamina propria of the former and the lower layer and muscle layer of the latter [15]. Th17 cells mainly exert proinflammatory and excessive immune activation roles in the pathogenesis of IBD. Th17 cells furthermore possess some protective qualities. To stop bacterial infection, lessen intestinal inflammation, repair tissue damage, and maintain the integrity of the intestinal barrier, their secreted IL-22 stimulates intestinal epithelial cell proliferation, improves goblet cell function and mucus production, and encourages the production of nucleotide-binding oligomerization domain-containing protein 2 [16]. However, this protective effect is significantly weaker in IBD patients compared with the proinflammatory effect [6]. Th17 cells have many roles in the development of IBD, and they can release the cytokines IL-17, IL-22, and IL-23 that draw neutrophils into the inflammatory process and exacerbate it. In a study, it was discovered that individuals with active IBD had considerably higher levels of Th17 cells, IL-17, and IL-21 expression, which was consistent with disease activity and mucosal injury [17]. In addition, intestinal fibrosis is a common finding in patients with IBD, which can lead to progressive tissue deformation, loss of function, and stenosis of the intestine. IL-17, on the other hand, also plays a great role in it. A recent study showed that anti-IL-17 antibody treatment significantly reduced colorectal fibrosis in colitic mice by downregulating the expression of collagen 3 and several profibrotic cytokines [18]. IL-23 is also an important cytokine, and a study assessing IL-23 expression in human IBD showed that IL-23 production by lamina propria macrophages was increased in CD patients. Recent research has revealed that IL-23 plays a critical role in the modulation of tissue inflammation in IBD and colitis-associated colon cancer [19, 20]. In addition to the Th17 cells acting on IBD through cytokines, there are other influencing factors. Endogenous gut microbiota is involved in Th17 differentiation when the gut microbiota is stable. Studies have confirmed that the intestinal microbiota promotes Th17 cells expansion in areas of intestinal inflammation in mice with experimental colitis with high infiltration of Th17 cells. A study found that *E. coli*/IL-35-treated mice showed fewer CD4+ IL-17A+ Th17 cells and that disease activity index (DAI), intestinal shortening, and histopathological changes were significantly reduced [21, 22]. In another study by Huang and colleagues, it was found that the supernatant of *F. prowazekii* improved and prevented colitis in mice by inhibiting Th17 differentiation and IL-17A secretion in plasma and colonic mucosal tissues, downregulating IL-6, and upregulating IL-4 [23]. Treg cells are potent T cells, in contrast to Th17 cells. Peripheral blood and colon mucosa of IBD patients contained fewer Treg cells, suggesting that a lack of Treg cells plays a role in the pathogenesis of IBD [15]. According to the study, IL-10 and TGF-*β* control Treg cells and the substances they produce, which prevent the intestinal inflammatory cascade from occurring. Additionally, essential routes for IBD recovery include the encouragement of tissue repair, cytolysis of effector cells, metabolic degradation, and inhibitory cytokine secretion [17, 24]. Inflammation of the midgut mucosa in IBD is important in maintaining and promoting the development of colorectal cancer, and Treg cells can effectively prevent and delay inflammation-mediated tumor growth by exerting antitumor activity [25]. In conclusion, Treg cells not only control intestinal inflammation but also suppress the development of autoimmunity.

The preservation of the mucosal immune system's proper functioning depends on the balance of immune cells in the intestinal mucosa and luminal contents. Th17 and Treg cells normally compete with one another to prevent the other from proliferating and maintain equilibrium. They also play a significant role in controlling T cell-mediated immune responses and reducing intestinal inflammation, and their dysregulation is likely to contribute to the pathogenesis of IBD [26–28]. The proinflammatory effect of Th17 cells causes a serious immune and inflammatory response that outweighs the immune tolerance and protection of Treg cells, which accelerates the progression of IBD. In the IBD state, a number of pathogens penetrate the intestinal epithelial barrier, activate antigen-presenting cells, and induce them to secrete inflammatory cytokines [29]. Numerous studies have also further corroborated that affecting the Th17/Treg balance can lead to the development of IBD. IBD is an independent risk factor for colorectal cancer development [30]. Upregulation of genes associated with Th17/Treg (IL-10, IL-23, and transcription factor Foxp3) was demonstrated to be critical for colorectal cancer development in Velikova's study [25]. In a number of inflammatory illnesses, the balance of Th17 and Treg cells controls the host immune response. This equilibrium is also intimately tied to intestinal microecology. According to a study, intestinal bile acid metabolites control the balance and operation of Th17 and Treg cells. The intrinsic mechanism is that 3-oxoLCA inhibits Th17 cells differentiation by directly binding to its key transcription factor ROR*γ*t and that isoalloLCA enhances Treg differentiation by producing mitochondrial reactive oxygen species, resulting in increased Foxp3 expression [31]. The immunological balance of the IBD colonic mucosa is significantly influenced by Th subsets, particularly Th17 cells and Treg cells. According to an investigation, miR-155 is overexpressed in the colonic mucosa of people with IBD, and miR-155 inhibitors can boost Treg cells and decrease Th17 cells, suggesting that miR-155 relieves colitis by balancing the Th17/Treg ratio. Consequently, the development of IBD therapeutics depends significantly on the balance between Th17 and Treg cells [32]. In summary, the mechanism of Th17/Treg cells balance in IBD does deserve our research attention.

## 4. Chinese Medicine Treatment Strategy of Balancing Regulation to Prevent and Treat IBD

**Figure 1:**
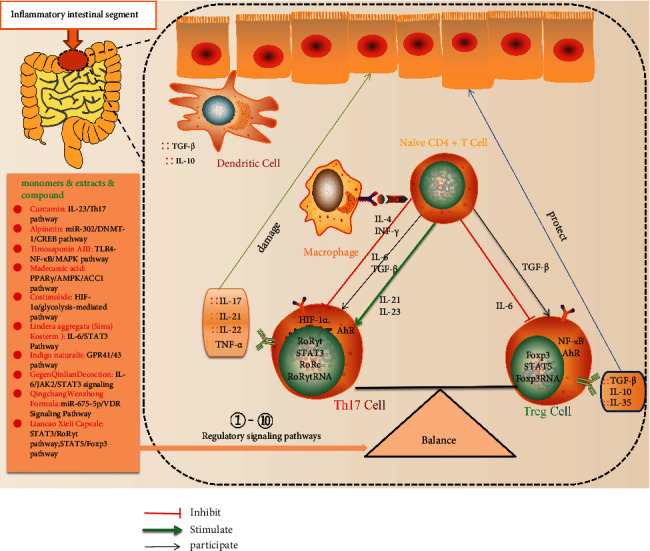
Mechanism of action of the monomers and compound formulations of traditional Chinese medicine. Under normal conditions, the intestine does not produce IL-6 due to the lack of pathogen stimulation from antigen-presenting cells, and naïve CD4+ T cells mainly differentiate into Treg cells under TGF-*β* to prevent the occurrence of autoimmune disease. When pathogens invade, antigen-presenting cells can secrete IL-6, and IL-6 acts together with TGF-*β* to induce the differentiation of naïve CD4+ T cells to Th17 cells and to inhibit the induction effect of TGF-*β* on Treg, with an enhanced immune response, contributing to the elimination of pathogens. When the inflammatory effect of Th17 cells exceeds the tolerance effect of Treg cells, the excess cytokines (IL-17, IL-21, IL-22, etc.) act on intestinal epithelial cells, macrophages, and colon myofibroblasts, induce proinflammatory chemokines and other proinflammatory mediators, mediate the early mobilization of granulocytes, and participate in the early inflammatory mediator response, significantly greater than the immune protective effect of cytokines secreted by Treg cells (TGF-*β*, IL-10, etc.); the body is prone to induce IBD. In this paper, the TCM monomer, extract, and compound (such as *Abelmoschus manihot*, Daphnetin, epigallocatechin-3-gallate, Baitouweng Decoction, and compound sophorae decoction) are exactly the different stages of the differentiation and regulation of Th17 cells and Treg cells, thus inhibiting the differentiation and production of Th17 cells, reducing the number of associated damage factors that it has secreted, reducing the inhibition of Treg cells differentiation and promoting its production, increasing the number of associated protective factors it secreted, further resulting in restoration of the intestinal immune balance, achieving therapeutic purposes.*Abbreviations*. AhR: aryl hydrocarbon receptor; AMPK: adenosine 5′-monophosphate- (AMP-) activated protein kinase; ACC1: acetyl-CoA carboxylase 1; CYP1A1: cytochromeP4501A1; CREB: cAMP-response element binding protein; DC: dendritic cells; DNMT-1: DNA methyltransferase-1; Foxp3: forkhead-like protein 3; GPR41: G-protein receptor 41; HIF-1*α*: hypoxia-inducible factor 1-alpha; IL: interleukin; miR-302: microRNA-302; MAPK: mitogen-activated protein kinase; NF-*κ*B: nuclear factor kappa-B; PPAR*γ*: peroxisome proliferator-activated receptor *γ*; PHD2: prolyl hydroxylase 2; ROR*γ*t: receptor-associated orphan receptor *γ*t; RORC: receptor-associated orphan receptor; STAT3: transcription protein 3; STAT3: transcription protein 3; TGF: transforming growth factor; TLR4: toll-like receptor 4.

Due to its traits of having many targets, multiple approaches, high safety, and a low price, TCM provides distinct advantages in the treatment of IBD. With the advancement of contemporary therapy, studies have confirmed that TCM monomers, as well as active ingredients, TCM compounds, and TCM preparations, have a regulatory effect on Th17/Treg balance in IBD. Its mechanism of action has also been presented in the form of pictures (Figure 1). It can be used as an alternative treatment for IBD and has reference value.

### 4.1. Chinese Herbal Monomers and Their Extracts Prevent and Treat IBD by Regulating Th17/Treg Balance

Studies on the pathogenesis of IBD show that Th17/Treg imbalance is critical, and on this basis, there are also many studies on the treatment of IBD by TCM and its extracts by regulating Th17/Treg imbalance in the body. Paeoniflorin (PF) is a monoterpene glycoside component extracted from the roots of *Paeonia lactiflora* Pall. It has analgesic, immunomodulatory, and antitumor pharmacological effects [33–35]. A study found that PF can reduce histological scores and alleviate the disease by regulating the Th17/Treg balance mediated by dendritic cells [36]. In addition, PF can also significantly downregulate IL-17 and increase IL-10 levels in experimental colitis models, indicating that PF has the property of reducing inflammatory infiltration [37]. The lipophilic polyphenol curcumin, which is produced from the *Curcuma longa* root, has its own anti-inflammatory and anti-infective properties [38, 39]. A study showed that curcumin increased the level of cytokine IL-10 by inhibiting the IL-23/Th17 pathway, thereby restoring the balance of Treg/Th17 while reducing the concentration of proinflammatory cytokines. This further reduced the inflammatory response, reducing intestinal endothelial cell swelling, increasing intestinal mucosal permeability, and achieving a therapeutic effect in reducing intestinal inflammation as a whole [40]. 6-Gingerol is an extract of the rhizome of *Zingiber officinale* Roscoe, which has a number of pharmacological effects, for instance, antioxidation, antitumor, and enhancing immunity [41, 42]. According to a study, 6-gingerol significantly reduced intestinal damage and pathological inflammatory cell infiltration by inhibiting the upregulation of the transcription factor ROR*γ*t and the downregulation of the Treg transcription factor Foxp3 in Th17 cells. It also inhibited the increase and decrease in the number of Th17 cells and Treg cells, restoring the balance between the two cell types. Additionally, excessive IL-6 expression leads to an imbalance in the development and functionality of Th17 and Treg cells. 6-Gingerol works by inhibiting IL-6 expression and secretion to maintain the proper ratio of Th17 to Treg cells [43, 44]. An active ingredient derived from *Tripterygium wilfordii* Hook F., *Tripterygium wilfordii* polyglycoside, possesses antitumor, immunomodulatory, and other pharmacological properties [45, 46]. *Tripterygium wilfordii* polyglycoside was found to inhibit Th17 cells differentiation by downregulating RORC and STAT3 expression, while IL-17 production upregulated Foxp3 and promoted Treg cells formation, further regulating Th17/Treg balance and inhibiting intestinal inflammation in colitic mice [47]. Baicalein is a flavonoid bioactive component derived from *Scutellaria baicalensis*, and its pharmacological effects, such as antibacterial, neuroprotective, and antitumor, have been demonstrated in a variety of disease models [48, 49]. A study found that baicalein could maintain intestinal immune homeostasis and reduce intestinal inflammatory damage in mice with colitis. Its intrinsic mechanism of action is to promote Treg cells differentiation by activating the aryl hydrocarbon receptor (AhR) while also lowering IL-6 levels and increasing TGF-*β* levels in colitic mice to prevent the onset of Th17 cells differentiation, thereby regulating the balance of Th17/Treg cells and alleviating colitic mouse symptoms [50]. There is evidence that the bioactive compound berberine, which is derived from the Chinese herb *Coptis chinensis* Franch., has a diversity of pharmacological properties, including anti-inflammatory, antioxidant, antiatherosclerotic, antibacterial, antidiabetic, and neuroprotective benefits [51, 52]. Berberine can reduce DAI, colon shortening, and colon tissue damage in IBD by regulating Treg/Th17 balance. It has been found that berberine inhibits the differentiation of naïve CD4+ T cells into Th17 cells by downregulating the expression of TGF-*β*. Simultaneous activation of AhR transcription factors, by upregulating cytochromeP4501A1 levels, increases Foxp3 expression, causes Treg cells differentiation, and comprehensively regulates Th17/Treg balance through these two pathways [53–55]. Alpinetin is a flavonoid isolated from *Alpinia officinarum Hance*, which has hepatoprotective, cardiovascular, antibacterial, antiviral, and neuroprotective effects and is widely used in medicine [56, 57]. Studies have confirmed that Alpinetin promotes Treg cells differentiation and restores Th17/Treg balance by activating AhR, promoting miR-302 expression, while downregulating DNA methyltransferase-1 expression, reducing Foxp3 promoter region methylation levels, promoting cAMP-response element binding protein binding to Foxp3 promoter region, and upregulating Foxp3 expression, further improving intestinal mucosal pathological outcomes in colitic mice, including alleviating surface epithelial damage and significantly reducing crypt number and inflammatory cell infiltration [58]. *Abelmoschus manihot* is a TCM having pharmacological properties, including immunomodulation, cardioprotection, antidiabetic nephropathy, anticonvulsant, and antidepressant [59]. According to a study, *Abelmoschus manihot* increases the expression of Foxp3 and specific cytokines in Treg cells while decreasing the expression of RoR*γ*t and specific cytokines in Th17 cells, thereby balancing the expression of both to maintain intestinal immunological homeostasis [60]. The roots, barks, leaves, and flowers of *Daphne odora* Thunb. can all be used to extract Daphnetin. Modern pharmacological research has demonstrated that it has antioxidation, immunological modulation, and antitumor properties [61, 62]. Studies have shown that Daphnetin alleviates the symptoms of experimental colitis by alleviating colitis, improving colon injury, and reestablishing immune balance, and the intrinsic mechanism is that Daphnetin lessens the generation of Th17 cells while inhibiting the differentiation of Treg cells and restoring Th17/Treg balance [63]. Timosaponin AIII is one of the important active components extracted from *Anemarrhena asphodeloides* Bge., a TCM with a long history of medication, and most of its pharmacological effects are significant, while anticancer effects are potential [64, 65]. In a preliminary study by Lim et al. Timosaponin AIII could reduce NF-*κ*B activation as well as the levels of IL-1*β*, tumor necrosis factor-*alpha* (TNF-*α*), and IL-6 and increase IL-10 levels, and in addition, it was found that cellular differentiation of Th17 in the colonic lamina propria was inhibited, while Treg cells were induced, indicating that Timosaponin AIII inhibited colonic shortening and myeloperoxidase activity in mice by inhibiting activation of nuclear factor kappa-B (NF-*κ*B) and mitogen-activated protein kinase (MAPK) and restoring Th17/Treg balance for therapeutic purposes [66]. Poncirin is a flavonoid with extensive biological activities, including anticancer, antioxidant, antidiabetic, and other pharmacological effects [67]. A study showed that poncirin inhibited the differentiation of splenocytes into Th17 cells and the expression of IL-17 and Foxp3 *in vitro* and activated lipopolysaccharide- (LPS-) stimulated macrophages by inhibiting the binding of LPS to macrophage toll-like receptor 4. These increased the differentiation of splenocytes into Treg cells. Restoration of Th17/Treg imbalance through the above mechanisms suppresses NF-*κ*B activation, thereby alleviating colitis [68]. Green tea can be used to extract the naturally occurring substance epigallocatechin-3-gallate. In human physiology and pathophysiology, it plays a complex role. According to pharmacological research, it has antioxidation, anticancer, and antifibrosis properties [69]. A study showed that epigallocatechin-3-gallate reduced the induced expression of ROR*γ*t by suppressing IL-16 and inhibiting the expression of STAT3 and hypoxia-inducible factor -1alpha(HIF-1*α*) protein, thereby reducing Th17 cells production. In mice with experimental colitis, IL-6 downregulation reduces the amount of Treg cells it suppresses and eventually restores the Treg/Th17 ratio, which lowers DAI and spleen index and lessens colonic tissue erosion [70]. *Centella asiatica*, the dried whole plant, a member of the Umbelliferae family, was founded in *Shennong's Classic of Materia Medica* and is commonly used to treat various diseases of the digestive system, nervous system, and skin defense system. Madecassic acid is one of its main bioactive components, which has the effects of anticancer and anticancer [71]. Madecassic acid restores Th17/Treg balance by regulating the PPAR/AMPK/ACC1 pathway, resulting in anticolitis effects such as improved disease activity index, decreased colonic shortening, decreased myeloperoxidase activity, and decreased pathological damage in colitic mice colonic tissue [72]. One of the active ingredients in *Atractylodes macrocephala* Koidz. is costunolide, a naturally occurring sesquiterpene lactone, and pharmacological investigations have demonstrated that it has anticancer, anti-inflammatory, and antioxidant benefits [73, 74]. A study showed that oral administration of costunolide obviously improved DAI, rescued the reduction in colon length, and reduced proinflammatory cytokine levels in colitic mice. More in-depth mechanistic studies have shown that costunolide triggers the proteasomal degradation of the prolyl hydroxylase 2-triggered proline hydroxylation-ubiquitination-proteasome degradation of HIF-1*α*, inactivates the glycolytic process in Th17 cells, decreases the percentage of Th17 cells, and rebalances Th17/Treg in the colon and spleen [75]. Pharmacological studies have revealed that polydatin, one of the natural active ingredients isolated from the dried rhizomes of *Polygonum cuspidatum* Sieb., possesses asthmatic, antioxidant, and antitumor activities [76, 77]. As per studies, polydatin can successfully reduce the signs and symptoms of colitis in mice and protect and repair colonic mucosal tissues. This is done mostly by downregulating STAT3 signaling, inhibiting Th17 cell differentiation, thereby regulating the Th17/Treg balance [78]. A traditional Chinese herbal remedy called *Lindera aggregata (*Sims) Kosterm. has a diverse range of biological actions, including analgesic, antitumor, and antibacterial benefits. Studies have discovered that *Lindera aggregata (*Sims) Kosterm. can considerably reduce intestinal permeability and ameliorate colonic atrophy, both pathological alterations in colonic tissue that are common in UC model mice. The intrinsic mechanism of action is mainly to inhibit the production and secretion of IL-6, the transduction of the IL-6/STAT3 signaling pathway, and the polarization of Th17 *in vivo*, in order to adjust the balance of Th17 and Treg cells and ameliorate the symptoms and lesions of UC [79]. In clinical cases of inflammatory illnesses, cutaneous, mucosal, and other ailments, *Indigo naturalis* is frequently employed [80]. According to a study, Foxp3 and IL-10 expression were clearly up, ROR*γ*t and IL-17 expression were clearly down, and G protein-coupled receptor 41 (GPR41) and G protein-coupled receptor 43 (GPR43) protein expression were both noticeably higher in the colon of rats in the Qingdai group. Combined with the results of previous studies, it indicated that *Indigo naturalis* played a therapeutic role by affecting the GPR41/43 signaling pathway to regulate the downstream Th17/Treg balance [81] (Table 1).

### 4.2. Chinese Herbal Compound and Its Preparation Regulate Th17/Treg Cells Balance to Prevent and Treat IBD

Clinical trials and experimental studies have revealed that Th17/Treg imbalance has a correlation with the pathogenesis of IBD. Numerous studies of Chinese herbal compounds have also confirmed that intervention therapy with Chinese herbal compounds can regulate the imbalance status of Th17/Treg in IBD so as to achieve the effect of treating IBD. The main effects of these compounds are to clear heat and dampness, remove blood stasis and phlegm, and warm the intestine.

Gegen Qinlian Decoction (GQ), founded in the *Treatise on Febrile Diseases*, is a representative prescription for the treatment of clearing away heat and dampness to stop diarrhea and has been used in clinical settings for the long-term treatment of diarrhea and dysentery. Its ingredients include *Pueraria lobata* (Willd.), *Coptis chinensis* Franch., *Scutellaria baicalensis*, and *Glycyrrhiza uralensis* Fisch. [82]. Based on a study, administration of the GQ group significantly decreased the production of proinflammatory mediators, as well as the expression of inflammatory mediators like TGF-*β*1 and IL-17. It also inhibited the phosphorylation of JAK2 and STAT3, which in turn decreased STAT3's ability to regulate transcription. Inhibiting IL-6/JAK2/STAT3 signaling to reinstate Treg and Th17 homeostasis in colon tissue helped Gegen Qinlian Decoction reduce the sickness overall [83]. Qingchang Wenzhong Formula (QCWZ) has the effects of clearing away heat and dampness and stopping bleeding. *Coptis chinensis* Franch., *Ginger*, *Indigo naturalis*, *Sophora flavescens*, *Panax notoginseng*, Ulmus charcoal, and other drugs are the main ingredients [84]. A study found that, after treatment with QCWZ, the expression of miR-675-5p, ROR*γ*t mRNA in the colon, and IL-17 in the serum of UC rats was increased, while the expression of VDR mRNA, Foxp3 mRNA, ZO-1, IL-10 in serum was significantly decreased compared with the blank group, indicating that QCWZ targets and regulates the VDR signaling pathway by decreasing the expression of miR-675-5p. Thus, Th17/Treg balance is regulated to achieve the therapeutic purpose of repairing intestinal mucosal barrier injury in UC patients [85]. The composition of Shaoyao decoction (SY) includes *Paeonia lactiflora*, *Areca catechu* L, *Scutellaria baicalensis*, *Coptis chinensis, Angelica sinensis*, *Glycyrrhiza uralensis*, and *Aucklandia lappa* Decne. It is often used in clinical practice to treat abdominal pain of damp-heat type and hematochezia [86]. A study found that SY could reduce IL-17 and IL-23 in serum, ROR*γ*t, and HIF-1*α* levels in the colon and increase IL-10 in the serum of UC rats, indicating that SY treatment of UC rats may be achieved by inhibiting HIF-1*α* to regulate the rebalance of Treg/Th17 [87]. Baitouweng Decoction (BTW) is a representative formula in *Treatise on Febrile Diseases* for the treatment of pus and blood under the stool caused by heat toxin flaming, which is widely used and has an effect on various enteritis. Its composition mainly includes four herbs: *Pulsatilla chinensis*, *Coptis chinensis*, *Phellodendron amurense*, and *Cortex Fraxini* [88]. BTW intervention decreased the expression of cytokines associated with Th17 cells differentiation (including IL-6, IL-1*β*, and TNF-*α*) and upregulated the expression of IL-10, a key indicator of Treg cells differentiation, thereby regulating the proportion of Th17 cells and Treg cells in mice and restoring Th17/Treg balance, in order to reduce DAI scores and colonic pathological damage in mice [89]. The ingredients of Jiedu Huayu Decoction (JDHY), a TCM remedy for blood circulation improvement and stasis removal, include *Pulsatilla chinensis*, *Scutellaria barbata*, *Phellodendron amurense*, *Portulaca oleracea* L., and *Coptis chinensis*. Dandan Li and colleagues discovered that JDHY could improve the clinical symptoms of active UC patients with dampness-heat accumulation by lowering IL-17 levels, increasing IL-10 and TGF-*β* levels, regulating the expression of ROR*γ*t and Foxp3 mRNA, promoting the recovery of Th17/Treg balance, and lowering the recurrence rate [90]. Compound sophorae decoction (CS) is a TCM formula composed of *Sophora flavescens*, *Indigo naturalis*, *Glycyrrhiza uralensis*, and *Panax notoginseng* powder. In the spleen and mesenteric lymph nodes of mice, a focused investigation discovered that CS could decrease the proportion of Th17 cells and raise the fraction of Treg cells. And the fundamental mechanism is to alter the Th17/Treg balance in mice in order to improve the relevant illness markers [91]. Rhubarb Peony Decoction (RP), from the classical ancient book of TCM *Jin Gui Yao Lue*, has the effect of purging heat and breaking and dissipating swelling. RP consists of *Rhei Radix et Rhizoma*, *Moutan Cortex*, and *Persicae Semen*. A study found that RP reduced the proportion of Th17 cells in colon tissue, increased the proportion of Treg cells, promoted the recovery of Th17/Treg balance, assisted mice to reduce pathological changes, and improved weight loss and tissue damage [92]. Bawei Xileisan (BWXL) is a traditional remedy for clearing away heat and toxic substances, relieving swelling, and relieving pain. In the past, diphtheria was primarily treated with it. In modern therapeutic practice, it can be utilized to heal ulcers and colonic inflammation [93]. In accordance with a study, BWXL enema improved dramatically the structure of the shortened colon and the damaged tissue. The mechanism is that the expression levels of Th17-related cytokines IL-17 A/F and IL-22 are significantly and dose-dependently reduced, resulting in restoration of the Th17/Treg balance [94]. The Yi-Yi-Fu-Zi-Bai-Jiang-San (YYFZBJ) prescription and syndrome take into account numerous elements, like cold, dampness, heat, blood stasis, yang insufficiency, and spleen deficiency. These factors have the impact of tonifying deficiency and purging excess, as well as controlling cold and heat [95]. Thus according to studies, YYFZBJ contains coix seed, aconite root, and *Patrinia* and has good efficacy and an antitumor function. Thus, according to studies, YYFZBJ contains coix seed, aconite root, and *Patrinia* and has good efficacy and an antitumor function. A study indicated that YYFZBJ reduced the expression of ROR*γ*t mRNA in colon tissue and the level of IL-17 in blood, increased the expression of Foxp3 mRNA and the level of IL-10, and further affected the numbers of Th17 and Treg cells to restore the Treg/Th17 balance. This balance has a therapeutic impact [96]. The effects of the Qingchang Huashi Recipe (QCHS) involve clearing heat and dampness, balancing qi and blood, and chilling blood to prevent dysentery. *Astragalus membranaceus*, *Radix Paeoniae Alba*, *Pulsatillae Radix*, *Angelicae Dahuricae Radix*, and *Scutellariae Radix* are among the herbs that make up this composition. The study found that QCHS could reduce the DAI score and improve pathological injury in mice, especially in the high dose group of QCHS, which had a significant therapeutic effect, and its mechanism may be through downregulating IDO1 expression, downregulating ROR*γ*t and related proinflammatory factors, and upregulating Foxp3 and related anti-inflammatory factors, so as to maintain the balance of Th17/Treg cytokine expression in order to achieve a therapeutic effect [97]. Huangqin Decoction (HQ) is frequently used in the treatment of IBD in clinical practice, and this study found that it could reduce the expression of Th17 cytokines IL-17, IL-6 protein and mRNA, and transcription factor ROR*γ*t protein while increasing the expression of Treg cytokines IL-10, TGF-*β* protein and mRNA, and the protein level of transcription factor Foxp3 in colitis rats. The results suggest that HQ can balance the imbalance of Th17/Treg through the regulation of cytokines and transcription factors, which may be the immune mechanism by which HQ exerts its effect and protects the intestinal mucosa [98]. Liancao Xieli Capsule (LCXL) is used as a TCM compound preparation in the treatment of IBD. A study found that LCXL can reduce the expression levels of p-STAT3 and ROR*γ*t proteins in the colon tissue of mice and increase the expression levels of p-STAT5 and Foxp3 proteins. The specific therapeutic effect demonstrated that body weight, colon length, and TGF-*β* content in mouse colon tissue were significantly increased, while DAI score, colon histopathological score, and IL-6 and IL-17A content were significantly reduced. The mechanism of regulating Treg/Th17 immune balance to improve intestinal inflammation in mice is mainly through inhibiting STAT3/ROR*γ*t and promoting the activation of the STAT5/Foxp3 signaling pathway [99]. Wei Chang An Pill (WCA) is a TCM preparation with the effects of aromatizing turbidity, regulating qi, relieving pain, strengthening the stomach, and guiding stagnation. It can repair the intestinal mucosal injury and relieve clinical symptoms. According to Tao Zhang's study, different doses of WCA had distinct effects on the mouse colon tissues' Treg/Th17 levels. To regulate both percentages and regain the proper balance between them, the therapeutic effect was obtained by upregulating Treg cell percentage and downregulating Th17 cell percentage [100]. Kaijieling (KJL) is a TCM formula preparation that can invigorate the spleen, clear away heat, and promote blood circulation. Ingredients in KJL include *Atractylodes macrocephala*, *Paeonia lactiflora* L., *Hirudo*, and *Glycyrrhizauralensis*. The results demonstrated that KJL modulated the associated cytokines and transcription factors to increase Treg cells and inhibit Th17 cells, alleviating the pathological damage to the colonic mucosa and lowering the histological score following treatment. In order to counteract the imbalance between Th17 and Treg cells, it also suppresses the STAT3 pathway. Another study further supported the hypothesis that KJL's therapeutic effect may be due to a decrease in CD4+ IL-17A+ Th17 cells and an increase in CD4+ Foxp3+ Treg cells in peripheral blood, which in turn restores the body's Treg/Th17 balance [101–103]. Xiaokui Recipe (XK) is composed of *Pulsatilla chinensis*, *Punica granatum* L., *Lilii Bulbus*, *Galla chinensis*, *Tribulus terrestris*, *Coptis chinensis*, and other TCMs. Huijian Zhang's study showed that it could reduce the concentration of proinflammatory cytokines IL-17 and IL-6, increase the concentration of IL-10 and TGF-*β*, increase the expression of Foxp3, decrease the expression of ROR*γ*t, achieve the effect of promoting ulcer healing, indicating that XK could promote the differentiation and production of Treg cells in the colonic tissue of colitis rats, mediate the degree of immune tolerance, restrain the differentiation of Th17 cells to regulate Th17/Treg balance, maintain the immune balance *in vivo*, and reduce the intestinal inflammatory response, thus playing a therapeutic role [104]. Ulcerated enema is composed of *Lonicera caprifolium* and *Lilii Bulbus*. Intestinal fluid from ulceration enema (UE) may be therapeutic by regulating the Treg/Th17 immune balance in UC mice, specifically by increasing the number of Treg cells and upregulating IL-10 levels to reduce the number of Th17 cells. It has the effects of clearing away heat and dampness, protecting the membrane, and generating muscle [105] (Table 2).

## 5. Conclusions and Perspectives

Currently, a substantial body of research points to the dysregulated Th17/Treg balance as a key factor in the emergence of IBD. In patients with IBD, maintaining Th17/Treg balance is crucial for lowering intestinal mucosal inflammation, reliving clinical symptoms, and enhancing prognosis. We should investigate how TCM regulates the Th17/Treg balance in IBD because it has several targets and multiple regulatory mechanisms, is less toxic, and has reasonable R&D and usage costs. In order to find new complementary and alternative therapies from the core of IBD in combination with various disciplines, domestic and foreign scholars are actively conducting a large number of studies, collecting and screening the effective components and compounds of TCM, and doing so under the guiding advantages of TCM syndrome differentiation and treatment and overall concept. Although the paper explains the mechanism of action of TCM in regulating Th17/Treg balance to reduce the intestinal inflammatory response and repair intestinal mucosa in IBD patients, promoting intestinal immune response to protect intestinal mucosa, and improving clinical symptoms, it also provides a reference for the prevention and treatment of IBD by TCM. However, the pathogenesis of IBD, the composition of TCM ingredients, and TCM compounds are very complex, and the mechanism of action is not clear enough. The mechanism of action of TCM intervention in the treatment of IBD cannot be completely explained by regulating Th17/Treg balance, and it is necessary to use modern technologies such as systems biology and molecular informatics from multiple angles and interdisciplines for extensive and in-depth research and, at the same time, carry out some high-quality clinical randomized controlled experiments as soon as possible to break the limitations mainly based on basic research, which will not only improve the level of evidence but also help enrich the understanding of the role of TCM in the treatment of IBD and guide further achievement transformation and clinical medication reference.

## Figures and Tables

**Table 1:** Interventional effect of Chinese herbal monomers and Chinese herbal components on Th17/Treg balance in IBD.

Name	Th17/Treg balance mechanisms	Model	References
Paeoniflorin	By regulating DC	TNBS mice	[36, 37]
Curcumin	Regulated by inhibition of IL-23/Th17 pathway	DSS mice	[40]
6-Gingerol	Suppression of upregulation of the Th17 cells transcription factor ROR*γ*t and downregulation of the Treg cells transcription factor Foxp3	DSS mice	[43, 44]
*Tripterygium wilfordii* polyglycoside	Downregulation of RORC and STAT3 expression inhibited Th17 cells differentiation, upregulated Foxp3, and promoted Treg cells formation	TNBS mice	[47]
Baicalein	Activation of AhR promotes Treg cells differentiation, decreases IL-6, and increases TGF-*β* to impede the onset of Th17 cells differentiation	DSS mice	[50]
Berberine	Downregulation of TGF-*β* expression inhibits the differentiation of naïve CD4+ T cells into Th17 cells and activates AhR transcription factors, which cause Treg cell differentiation by upregulating CYP1A1 and increasing Foxp3 expression	DSS mice	[53–55]
Alpinetin	Activation of AhR, promoting miR-302 expression, simultaneous downregulating DNMT-1 expression, decreased methylation levels of Foxp3 promoter region, promoting CREB binding to Foxp3 promoter region, and upregulated expression of Foxp3, thus promoting Treg cells differentiation	DSS mice	[58]
*Abelmoschus manihot*	Reduce the expression of the Th17 cells transcription factor ROR*γ*t and enhance the expression of the Treg cells transcription factor Foxp3	DSS mice	[60]
Daphnetin	Reduce the generation of Th17 cells and inhibit the differentiation of Treg cells	DSS mice	[63]
Timosaponin AIII	By inhibiting TLR4-NF-*κ*B/MAPK signaling pathway	TNBS mice	[66]
Poncirin	Inhibit the differentiation of splenocytes into Th17 cells and increase the differentiation of splenocytes into Treg cells	TNBS mice	[68]
Epigallocatechin-3-gallate	Inhibition of STAT3 and HIF-1*α* protein expression decreased ROR*γ*t expression, thereby reducing Th17 cell production. Downregulation of IL-6 reduces its suppression of Treg cells	DSS mice	[70]
Madecassic acid	Regulate PPAR*γ*/AMPK/ACC1 pathway to restore	DSS mice	[72]
Costunolide	Trigger the PHD2-triggered proline hydroxylation-ubiquitination-proteasome degradation of HIF-1*α*, inactivate the glycolytic process in Th17 cells, and decrease the percentage of Th17 cells	DSS mice	[75]
Polydatin	Downregulate the STAT3 signaling pathway and inhibit Th17 cells differentiation	DSS and TNBS mice	[78]
*Lindera aggregata *(Sims) Kosterm.	Inhibition of IL-6 production and secretion, transduction of the IL-6/STAT3 signaling pathway, and polarization of Th17	DSS mice	[79]
*Indigo naturalis*	Regulated by affecting the GPR41/43 signaling pathway	DSS rat	[81]

*Abbreviations*. AhR: aryl hydrocarbon receptor; AMPK: adenosine 5′-monophosphate- (AMP-) activated protein kinase; ACC1: acetyl-CoA carboxylase 1; CYP1A1: cytochromeP4501A1; CREB: cAMP-response element binding protein; DC: dendritic cells; DNMT-1: DNA methyltransferase-1; Foxp3: forkhead-like protein 3; GPR41: G protein-coupled receptor 41; HIF-1*α*: hypoxia-inducible factor -1alpha; IL: interleukin; miR-302: microRNA-302; MAPK: mitogen-activated protein kinase; NF-*κ*B: nuclear factor kappa-B; PPAR*γ*: peroxisome proliferator-activated receptor *γ*; PHD2: prolyl hydroxylase 2; ROR*γ*t: retinoic acid-related orphan receptor gamm; RORC:retinoic acid-related orphan receptor; STAT3: transcription protein 3; STAT3: transcription protein 3; TGF: transforming growth factor; TLR4: toll-like receptor 4.

**Table 2:** Interventional effect of Chinese herbal compound on Th17/Treg balance in IBD.

Name	Equilibrium mechanism	Model	References
Gegen Qinlian Decoction	Restored by inhibition of IL-6/JAK2/STAT3 signaling	DSS rat	[83]
Qingchang Wenzhong Formula	Regulated by decreasing the expression of miR-675-5p and targeting and regulating the VDR signaling pathway	DSS rat	[85]
Shaoyao Decoction	Regulated by inhibiting HIF-1*α*	TNBS rat	[87]
Baitouweng Decoction	Decrease the expression of cytokines associated with Th17 cells differentiation (including IL-6, IL-1*β*, and TNF-*α*) and upregulate the expression of IL-10	DSS mice	[89]
Jiedu Huayu Decoction	Decrease IL-17, increase IL-10 and TGF-*β*, and regulate the expression of ROR*γ*t and Foxp3 mRNA	Patients	[90]
Compound Kushen Decoction	Decrease the proportion of Th17 cells and increase the proportion of Treg cells	DSS mice	[91]
Rhubarb Peony Decoction	Decrease the proportion of Th17 cells and increase the proportion of Treg cells	DSS mice	[92]
Bawei Xileisan	Expression of Th17-associated cytokines IL-17 A/F and IL-22 was decreased in a dose-dependent manner	DSS mice	[94]
Yi-Yi-Fu-Zi-Bai-Jiang-San	Decrease the expression of ROR*γ*tmRNA and the content of IL-17, increase the expression of Foxp3 mRNA, and increase the content of IL-10	TNBS rat	[96]
Qingchang Huashi Recipe	Downregulate IDO1 expression, ROR*γ*t, and related proinflammatory factors and upregulate Foxp3 and related anti-inflammatory factors	TNBS mice	[97]
Huangqin Decoction	Restoration of balance through regulatory effects on Th17 and Treg cytokines and transcription factors	TNBS rat	[98]
Liancao Xieli Capsule	Decrease the expression levels of p-STAT3 and ROR*γ*t proteins and increase the expression levels of p-STAT5 and Foxp3 proteins	DSS mice	[99]
Wei Chang An Pill	Increasing the percentage of Treg cells and downregulating the percentage of Th17 cells	DSS mice	[100]
Kaijieling	Inhibit the STAT3 pathway	TNBS rat	[101–103]
Xiaokui Recipe	Promote Treg cells differentiation and generation and inhibit Th17 cells differentiation	TNBS rat	[104]
Ulcerated enema	Increase the number of Treg cells, upregulate IL-10, and reduce the number of Th17 cells	DSS mice	[105]

*Abbreviations*. Foxp3: forkhead-like protein 3; HIF-1*α*: hypoxia-inducible factor -1alpha; IL: interleukin; IDO1: indoleamine 2,3-dioxygenase 1; JAK2: Janus kinase-2; miR-675-5pa: microRNA-675-5pa; ROR*γ*t: retinoic acid-related orphan receptor gamma t; STAT3: transcription protein 3; TGF; transforming growth factor; VDR: vitamin D receptor; TNF-α: tumor necrosis factor -alpha.
